# Biphasic Effect of Buspirone on the H-Reflex in Acute Spinal Decerebrated Mice

**DOI:** 10.3389/fncel.2019.00573

**Published:** 2020-01-15

**Authors:** Yann Develle, Hugues Leblond

**Affiliations:** Department of Anatomy, CogNAC Research Group, Université du Québec à Trois-Rivières, Trois-Rivières, QC, Canada

**Keywords:** serotonin, 5-HT_1A_ receptor agonist, spinal cord injury, locomotion, sensorimotor

## Abstract

Pharmacological treatment facilitating locomotor expression will also have some effects on reflex expression through the modulation of spinal circuitry. Buspirone, a partial serotonin receptor agonist (5-HT_1__A_), was recently shown to facilitate and even trigger locomotor movements in mice after complete spinal lesion (Tx). Here, we studied its effect on the H-reflex after acute Tx in adult mice. To avoid possible impacts of anesthetics on H-reflex depression, experiments were performed after decerebration in un-anesthetized mice (*N* = 20). The H-reflex in plantar muscles of the hind paw was recorded after tibial nerve stimulation 2 h after Tx at the 8th thoracic vertebrae and was compared before and every 10 min after buspirone (8 mg/kg, i.p.) for 60 min (*N* = 8). Frequency-dependent depression (FDD) of the H-reflex was assessed before and 60 min after buspirone. Before buspirone, a stable H-reflex could be elicited in acute spinal mice and FDD of the H-reflex was observed at 5 and 10 Hz relative to 0.2 Hz, FDD was still present 60 min after buspirone. Early after buspirone, the H-reflex was significantly decreased to 69% of pre-treatment, it then increased significantly 30–60 min after treatment, reaching 170% 60 min after injection. This effect was not observed in a control group (saline, *N* = 5) and was blocked when a 5-HT_1__A_ antagonist (NAD-299) was administered with buspirone (*N* = 7). Altogether results suggest that the reported pro-locomotor effect of buspirone occurs at a time where there is a 5-HT_1__A_ receptors mediated reflex depression followed by a second phase marked by enhancement of reflex excitability.

## Introduction

During locomotion, afferent inputs from the hind limbs serve to control the excitability of spinal networks. They adjust motor output by direct impact on either motoneurons or interneurons, comprising those of the central pattern generator (CPG) that is responsible for locomotion (Rossignol, 2006). After complete spinal cord injury, sensory feedback becomes the only source of input remaining to the spinal cord, it has the power to re-arrange spinal circuits below the lesion, as shown by the positive outcome of treadmill training in adult cats (Lovely et al., 1986; Barbeau and Rossignol, 1987; Belanger et al., 1996), rats (Edgerton et al., 1997; Ichiyama et al., 2008; Otoshi et al., 2009), and mice (Leblond et al., 2003). The plasticity involved in this recovery of locomotion necessarily entails changes in several reflex pathways (Côté et al., 2003; Côté and Gossard, 2004).

In spinal animals, pharmacological treatments that mimic neurotransmitters from severed, descending fibers also have neuromodulator effects on locomotor networks and can improve recovery of locomotion (Chau et al., 1998a, b). As is the case with locomotor training, drugs that enable functional recovery also regulate spinal reflexes (Côté et al., 2003; Frigon et al., 2012). For example, in cats with complete spinal lesion, the noradrenergic agonist clonidine, which is known to trigger hind limb locomotion (Barbeau and Rossignol, 1987), was also found to modify spinal neuron responses to peripheral inputs (Barbeau and Rossignol, 1987; Chau et al., 1998a; Côté et al., 2003; Frigon et al., 2012). In rodents, serotoninergic (5-HT) drugs are effective in triggering and facilitating locomotion after complete spinal lesion (Schmidt and Jordan, 2000; Slawinska et al., 2014). Recent work in our laboratory has established that treatment with the US Food and Drug Administration-approved 5-HT_1__A_ receptor partial agonist buspirone (Loane and Politis, 2012) can initiate locomotion in the hind limbs of adult mice immediately after complete spinal lesion (Jeffrey-Gauthier et al., 2018). As drugs with pro-locomotor properties also modify reflex pathways, buspirone may alter reflex excitability in mice after complete spinal lesion.

The effects of 5-HT_1__A_ agonists on spinal reflexes have been tested earlier in different animal models, but there is still no consensus today as to whether the outcome is excitatory or inhibitory. On the one hand, *in vitro* results on isolated brainstem and spinal cord in neonatal rats indicate that buspirone decreases monosynaptic reflex excitability (Yomono et al., 1992). This observation concurs with other studies that have demonstrated 5-HT_1__A_ receptor inhibition in reflex pathways (Nagano et al., 1988; Crick et al., 1994; Hasegawa and Ono, 1996a, b; Honda and Ono, 1999). On the other hand, some have reported excitatory effects of 5-HT_1__A_ (Clarke et al., 1996), mainly by showing facilitatory effects on motoneuron depolarization (Takahashi and Berger, 1990; Zhang, 1991; Perrier et al., 2003; Grunnet et al., 2004) or monosynaptic reflex enhancement (Honda and Ono, 1999). Is it possible that substances with excitatory effects on locomotion also have inhibitory effects on spinal cord excitability?

The present study was performed with a newly developed model of decerebrated mice and was designed to investigate the modulation of reflex pathways in the absence of pharmacological anesthesia. This was required, since locomotion involves wide re-organization of reflex pathways, as shown mainly in decerebrated cat preparations in which new relays were described in the absence of anesthesia (McCrea, 2001). Some reflex pathways are thus state-dependent, meaning that they occur only when the CPG is driving locomotion or when drugs known to trigger locomotion are given (Gossard et al., 1994; Perreault et al., 1995; Leblond et al., 2000, 2001).

Here, the main objective is to assess the effect of buspirone, at a dose level that is known to trigger locomotion (Jeffrey-Gauthier et al., 2018), on H-reflex amplitude in adult decerebrated mice after acute spinal cord lesion. This reflex, the electrical analog of the tendon tap reflex, is primarily mediated by monosynaptic pathways (Misiaszek, 2003) and regroup both sensori- and motor systems. A second objective was to evaluate if the observed buspirone effect was mediated by 5-HT_1__A_ by blocking these receptors with the specific 5-HT_1__A_ antagonist NAD-299 (Johansson et al., 1997). The results show a biphasic effect of buspirone on the H-reflex: a significant decrease was first observed followed by an increase of the reflex 30 min later. Since buspirone had no effect if preceded by NAD-299, it is suggested that reflex modulation by buspirone is mediated by 5-HT_1__A_ receptors. Some of these results have been presented in abstract form Develle and Leblond (2016).

## Materials and Methods

### Animal Care and Ethics

Experiments were performed on 20 C57 mice, of either sex (Charles River Laboratories, Saint-Constant, QC, Canada), weighing 20–30 g. Their living conditions were strictly controlled by laboratory and facility staff. They were housed in cages with food and water available *ad libitum*. All manipulations and procedures were in accordance with Canadian Council on Animal Care guidelines and were approved by the Université du Québec à Trois-Rivières Animal Care Committee. The mice were randomly assigned to 1 of 3 groups in acute, terminal experiments to evaluate the effect of buspirone on the H-reflex: a group (*N* = 8) exposed to buspirone, a group (*N* = 7) exposed to 5-HT_1__A_ antagonist NAD-299 and buspirone, and controls (*N* = 5) treated with saline.

### Anesthesia

All surgeries were performed under isoflurane anesthesia (2% mixed with O_2_ 95% and CO_2_ 5%, 200 ml/min). General anesthesia was first induced through a mask: then, the animals were tracheotomized to maintain anesthesia and allow artificial ventilation (SAR-830/P Ventilator, CWE, Inc., Ardmore, PA, United States) adjusted to preserve expired CO_2_ level between 3 and 4% (CapStar-100 CO_2_ monitor, CWE, Inc.). Body temperature was monitored by rectal probe and maintained at 37 ± 0.5°C with heating pad.

### Spinalization

The objective was to measure the H-reflex after complete spinal cord section. It was performed early in the surgery to minimize the impact of the decerebration on the spinal circuitry. The paravertebral muscles were cleared from both vertebral laminae after skin incision targeting the 8th thoracic vertebra. Then, double laminectomy exposed the spinal cord at this level. After perforation of the dura mater with a needle, a small piece of lidocaine-soaked cotton (xylocaine 2%) was applied for 1 min to prevent uncontrolled secondary neural damage or lumbar spinal cord excitotoxicity. Then, the spinal cord was transected with micro-scissors and confirmed by visual observation of the gap between the rostral and caudal stumps. Finally, Surgicel^®^ absorbable hemostat (Ethicon, Johnson & Johnson, United States) was inserted between the two parts of the spinal cord before the skin was sutured.

### Decerebration

Spinal network activities are traditionally assessed in decerebrate preparations, especially from cats, rats and rabbits, with recent adaptation to mice (Dobson and Harris, 2012; Meehan et al., 2012, 2017). Data were, therefore, acquired in decerebrated, unanesthetized mice to avoid the unwanted effects of anesthesia. The carotid arteries were first ligated to minimize cerebral perfusion while the animals were secured in a stereotaxic frame (Model 980 Small Animal Spinal Unit, Kopf Instruments, Sunland-Tujunga, CA, United States) equipped with a small mouse and neonatal rat adaptor (Stoelting Company, Wood Dale, IL, United States). They were then craniotomized, taking care to leave the superior sagittal sinus intact. Bone wax (Ethicon, Johnson & Johnson, United States) was applied to the skull when necessary to prevent bleeding. The dura mater was removed gently to expose the cortex for transection with a razor blade 1 mm rostral to the lambda. The rostral part of neural tissue and the occipital cortex were removed, by gentle suctioning with an adapted micro-vacuum, corresponding to pre-collicular-pre-mamillar decerebration. The cavity was finally filled with Gelfoam^®^ thin soak hemostat sponge (Pfizer Inc., New York, NY, United States), and the skin was closed with suture.

### H-Reflex Recording

After decerebration, the left hind limb was fixed in extension and an incision was made on top of the gastrocnemius muscles to separate and expose the tibial nerve. A pool was formed with skin flaps and filled with mineral oil to avoid nerve desiccation. The tibial nerve was mounted on a home-made bipolar hook electrode for stimulation. One-ms single-pulsed stimulations were delivered by a constant-current stimulator (Model DS4, Digitimer Ltd., Welwyn Garden City, United Kingdom) triggered by a computer-controlled sequencer (Power 1401 acquisition system, Cambridge Electronic Design, Cambridge, United Kingdom).

Paired, fine, multistrained stainless steel wires (AS631Cooner Wire, Chatsworth, CA, United States) were inserted under the skin, between the second and third medial toes, toward the intrinsic foot muscles, for electromyographic (EMG) recording. Signals were amplified 1,000×, bandpass-filtered at 30–3,000 Hz (Grass P55 AC Preamplifier, Natus Neurology, Inc., Pleasanton, CA, United States), and digitized for data acquisition (Spike 2 software, Cambridge Electronic Design, Cambridge, United Kingdom). A ground electrode was inserted in the skin between the stimulating and recording electrodes.

Anesthesia was stopped, followed by 60-min rest, which corresponds to approximately 120–150 min post-spinalization, to avoid undesirable anesthesia-induced effects. Typically, mice can spontaneously move its forelimbs at this time but it should be noted that reflex recording was always made during a quiescent EMG background.

### Drug Administration

The H-reflex was compared between the three groups of mice: (1) buspirone only; (2) NAD-299 and buspirone; and (3) control. A catheter was inserted to facilitate i.p., administration without moving the animals. Buspirone (8 mg/kg, i.p.) was given in a volume of 0.1 cc of saline (0.9%) in the first group. This dose of buspirone was chosen since it was shown to trigger locomotion (Jeffrey-Gauthier et al., 2018). The second group received the 5-HT_1__A_ antagonist NAD-299 (0.66 mg/kg) (Johansson et al., 1997) 10 min before buspirone treatment. The third group received 0.1cc of saline only (0.9%).

### Data Acquisition and Analysis

**FIGURE 1:**
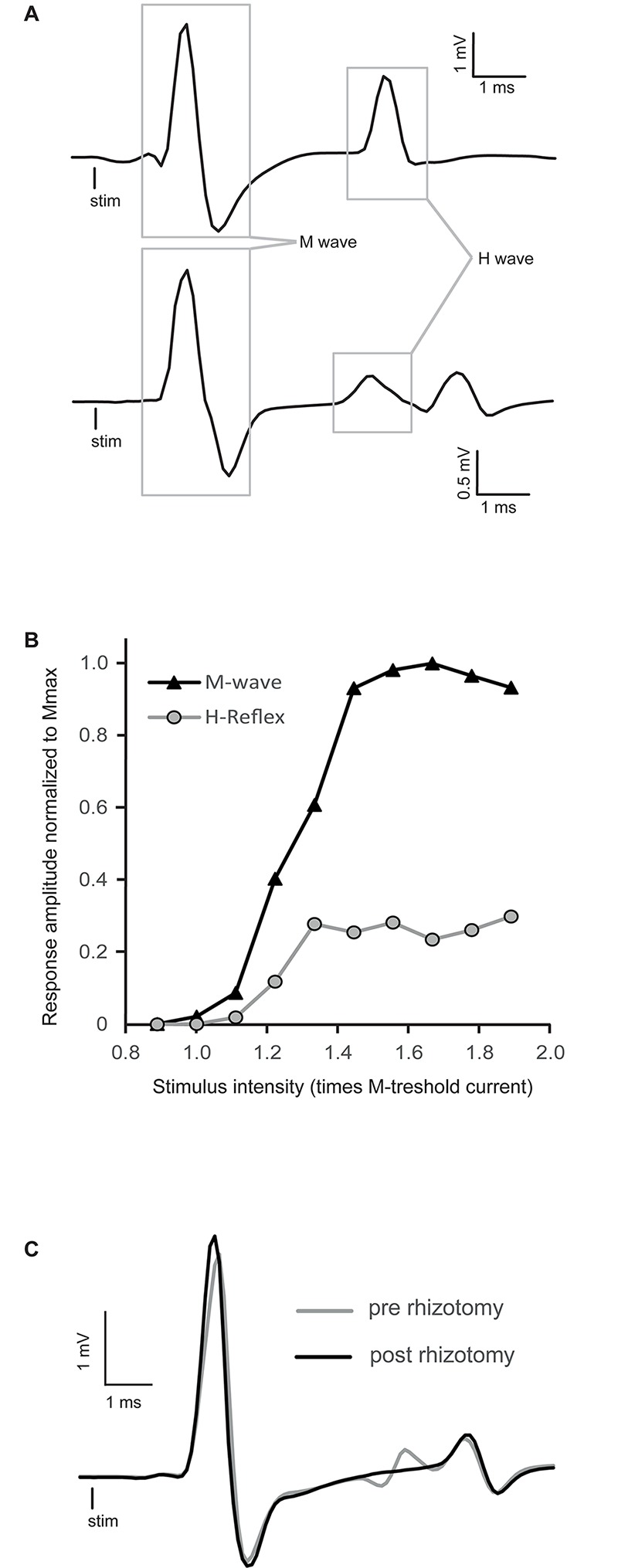
Raw traces of H-reflex and representative recruitment curve. **(A)** Averaged traces of electromyographic (EMG) recording showing typical examples of H-reflexes in decerebrated spinal mice. The M-wave is the depolarization of the whole motoneuron pool activated by stimulation, whereas the H-wave is the motor response induced by primary afferent depolarization. **(B)** Peak-to-peak amplitude of EMG responses recorded in intrinsic foot muscles by progressively increasing tibial nerve stimulation intensity. This stimulus–response curve is tested to find the stimulation intensity that will allow a stable reflex amplitude evaluation. It should be around Hmax, which specifically activates Ia primary afferents, concomitant with a stable M-wave response at the beginning of the M-wave plateau. In this particular example, stimulation around 1.4 times the motor threshold would be chosen. **(C)** Comparison of EMG traces of the H-reflex recorded before (gray trace) and after a complete dorsal rhizotomy (black trace). Sectioning all the dorsal roots at the lumbar enlargement abolished the H-reflex.

Stimulus–response curves (e.g., Figure 1B) were charted by gradually increasing tibial nerve stimulation intensity to ascertain the maximal H-reflex (4–6 ms latency) concomitant with stable M-wave (1–3 ms latency). At this intensity, which corresponded to approximately 1.8 times the motor threshold, Ia muscle spindle afferents were mainly activated. Responses to tibial nerve stimulation intensity were recorded before and every 10 min after the injection for a total of 60 min. Reflex amplitude was estimated with the H/M ratio which estimate the relative amount of motor neuron activated by the reflex loop as compared to the whole motor pool activated by the stimulation. This standardization allow a better inter-subject comparison of the reflex evolution in time and treatment.

Frequency-dependent depression was tested before and 60 min after injection in the buspirone treated mice by varying stimulation frequency between 4 blocks of 30 stimulations (0.2, 5, 10, and 0.2 Hz; 60 s inter-block interval). The first five responses of each block were discarded to allow H-reflex stabilization. Analyses comprised only recordings with stable M-wave throughout the protocol (<10% variation) to ensure recording stability.

Data were analyzed with Spike2 software (Cambridge Electronic Design) and Excel software (Microsoft Corporation, Redmond, WA, United States). Peak-to-peak amplitudes of the H-reflex and M-wave were measured to establish the H/M ratio so that the results could be compared between animals. Mean ratio at each time point was computed by averaging 30 stimulations at 0.2 Hz. In the frequency-dependent depression (FDD) protocol, mean H/M ratio was averaged from 25 successive responses for each block.

Statistical analysis was conducted with Statistica software (version 13, StatSoft Inc., Tulsa, OK, United States), and the significance threshold was set at *p* ≤ 0.05. Distribution’s normality was confirmed by the Kolmogorov–Smirnov test for each group separately. To be able to perform a balanced ANOVA, one missing sample at T60 in the group injected with saline, one in the group which receive both NAD-299 and buspirone and two in the buspirone treated group were replaced by the mean of the group for this timepoint. Then, Greenhouse-Geisser-corrected mixed ANOVA ascertained the effects of the intra-subject factor *time* and the inter-subject factor *treatment* on reflex amplitude. Fisher *post hoc* was used to observed periods presenting significant variations in comparison to pre-injection values. In the buspirone group, we further examined the impact of the frequency of stimulation (0.2, 5, and 10 Hz) on H/M ratio before (*T* = 0 min) and 60 min after (*T* = 60 min) buspirone injection. A repeated-measure ANOVA was used to verify the effects of frequency and buspirone and possible interaction (frequency × buspirone) on H/M ratio. When appropriate, effects were adjusted using the Greenhouse–Geisser correction.

## Results

### H-Reflex in Acute Spinal Decerebrated Mice

Stimulus–response curves were recorded for each mouse to establish at which intensity the H-reflex should be evoked to test the effect of buspirone. Typical examples of the H-reflex and stimulus–response curves are depicted in Figures 1A,B, respectively. Stimulation intensity was increased progressively until the whole pool of fibers in the tibial nerve was recruited, as indicated by a plateau being reached in the M-wave in Figure 1B. The H-reflex was usually evoked close to the motor threshold, and after an initial rise, it too plateaued and did not manifest a classical decrease in amplitude as it is observed in humans after reaching maximum (Knikou, 2008). This pattern was observed in all animals. Stimulation intensity was selected so that stable M-wave could be evoked as near as possible to beginning of the plateau (1.4T in the example depicted in Figure 1B).

As illustrated in Figure 1A (bottom trace), some responses included a third deflection with longer latency beginning about 7–8 ms post-stimulation. This late response was poorly depressed, if not depressed at all by stimulation frequency, in contrast to the response localized between 4 and 6 ms. For this reason this third deflection was not taken into account in the measurement of H-reflex. In one mouse, reflex recordings were made after a laminectomy and a complete lumbar dorsal rhizotomy to make sure that the first deflection was indeed the result of the activation of monosynaptic sensory inputs. By comparing the gray trace (pre-rhizotomy) and the black trace (post-rhizotomy) in Figure 1C, it is clear that the rhizotomy abolished the early components of the response and not the later responses. This indicates that this later response is not the result of segmental afferent inputs activation, since the dorsal roots are cut, and might be associated with antidromic muscle responses [namely F-wave (Meinck, 1976; Gozariu et al., 1998)].

### Buspirone Effect on the H-Reflex

**FIGURE 2:**
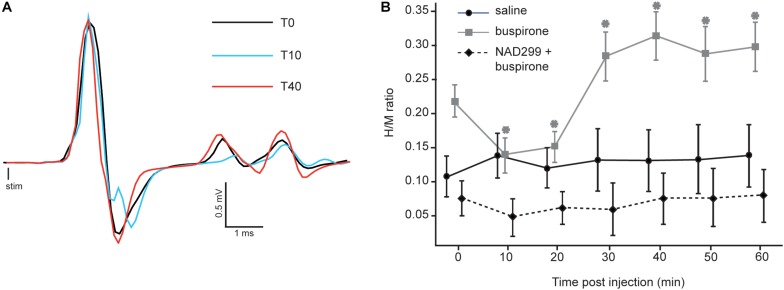
Effect of buspirone on the H-reflex. **(A)** Averaged EMG traces obtained at three critical time points after buspirone administration. The H-reflex amplitude shows an initial decrease 10 min after buspirone administration (blue trace) and an increase 40 min after buspirone (red trace). **(B)** Mean H/M ratios for mice from the three groups. For the buspirone group (gray line), the mean H-reflex is significantly lower than baseline at 10 and 20 min after buspirone whereas it is significantly higher than baseline at 30–40–50 and 60 min (^∗^*p* < 0.01). The amplitude of the mean H-reflex over time is no different than baseline in the saline group (black line) or the NAD-299 + buspirone group (dotted line).

Since buspirone can produce locomotion in chronic spinal mice (Jeffrey-Gauthier et al., 2018), we examined whether this behavioral effect could be related to changes in monosynaptic reflex excitability. Figure 2A displays H-reflex raw traces averaged from 30 stimulations recorded from intrinsic foot muscles before (black trace), 10 (blue trace) and 40 min (red trace) after buspirone administration in 1 mouse. Average H-reflex decreased dramatically in this mouse 10 min after buspirone, then increased considerably 40 min later.

The H/M ratio was measured for each mouse every 10 min for 60 min in each group (see Table 1). It was compared between the three groups using a mixed ANOVA to examine whether buspirone could change monosynaptic reflex excitability and whether this effect could be blocked by NAD-299, an antagonist of 5-HT_1__A_ receptors. Results indicate that reflex excitability was different between groups across time points (interaction: *F*_12_,_102_ = 4.4, *p* < 0.001; η^2^*_*p*_* = 0.34: see Figure 2B). Indeed, the buspirone group showed a biphasic change in monosynaptic reflex excitability with an early inhibition followed by facilitation. This effect was not observed for the saline group and was blocked when buspirone was administered after a treatment with the 5-HT_1__A_ antagonist NAD-299. Fisher *post hoc* test revealed that changes in reflex excitability were significant for each time point compared with T0 in the buspirone group (all *p* < 0.01). In contrast, no significant change was observed for any time point compared with baseline for the saline group (all *p* > 0.3) or for the NAD-299 + buspirone group (all *p* > 0.2).

**TABLE 1:** H/M ratio for each mouse.

**Time (min)**	**0**	**10**	**20**	**30**	**40**	**50**	**60**
**Buspirone (*N* = 8)**							
B1	0.350	0.287	0.302	0.463	0.494	0.460	0.510
B2	0.290	0.091	0.111	0.164	0.245	0.215	0.390
B3	0.272	0.131	0.128	0.365	0.398	0.303	0.365
B4	0.256	0.084	0.129	0.372	0.389	0.306	0.360
B5	0.205	0.281	0.186	0.391	0.257	0.554	0.312
B6	0.206	0.148	0.226	0.232	0.261	0.197	0.142
B7	0.140	0.116	0.142	0.272	0.409	0.216	0.216
B8	0.076	0.050	0.054	0.067	0.091	0.094	0.121
**NAD-299 + buspirone (*N* = 7)**							
BN1	0.058	0.042	0.064	0.058	0.065	0.079	0.077
BN2	0.037	0.016	0.034	0.026	0.040	0.034	0.038
BN3	0.076	0.050	0.078	0.029	0.103	0.114	0.116
BN4	0.055	0.008	0.009	0.033	0.040	0.041	0.035
BN5	0.039	0.036	0.039	0.050	0.041	0.029	0.039
BN6	0.163	0.120	0.151	0.160	0.169	0.171	0.167
BN7	0.093	0.053	0.046	0.055	0.060	0.063	0.074
**Saline (*N* = 5)**							
S1	0.124	0.143	0.077	0.104	0.068	0.090	0.110
S2	0.189	0.266	0.229	0.292	0.293	0.294	0.301
S3	0.124	0.127	0.133	0.111	0.120	0.124	0.116
S4	0.081	0.142	0.135	0.162	0.167	0.123	0.135
S5	0.069	0.054	0.071	0.034	0.050	0.077	0.069

*Peak to peak amplitude of H- and M-responses are compared using the H/M ratio which are given in this table for each mouse in all groups each 10 min for 60 min.*

### Frequency-Dependent Depression of the H-Reflex

**FIGURE 3:**
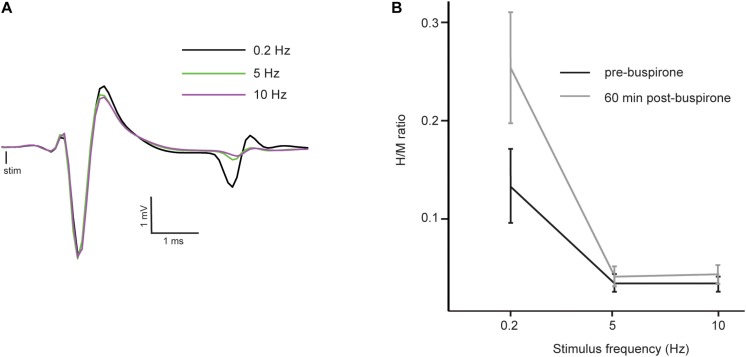
Reflex inhibition at 5 and 10 Hz. **(A)** Averaged EMG traces at 0.2 Hz (black trace), 5 Hz (green trace) and 10 Hz (purple trace) in a mouse before the administration of buspirone showing a typical example of depression of the H-reflex at higher frequency of stimulation (FDD). Consistency in fiber recruitment by stimulation is assessed by stable M-wave between each trial. **(B)** Mean H/M ratios at different stimulation frequencies in the buspirone group before (black line) and 60 min (gray line) after buspirone administration. The frequency significantly depressed the mean H/M ratio both before and after buspirone indicating that FDD is not abolished by the treatment.

The H-reflex is characterized by frequency-dependent behavior: as stimulation frequency increases from 0.2 to 10 Hz, the reflex amplitude is depressed. Figure 3A shows a typical example of FDD of the H-reflex in a mouse before buspirone treatment. In this example, frequency of stimulation at 5 Hz (green trace) or 10 Hz (purple trace) almost completely abolished the H-reflex that was observed at 0.2 Hz (black trace). In order to investigate if the observed late effect of buspirone on the H-reflex could be the result of a disinhibitory mechanism, FDD was thus compared at T0 (Figure 3B, black line) and 60 min after treatment (Figure 3B, gray line) in the buspirone group. Overall, H/M ratio was altered by the frequency of stimulation (principal effect of frequency: *F*_2_,_12_ = 17.6, *p* = 0.006, η_p_^2^ = 0.75) and by buspirone (principal effect of buspirone: *F*_1_,_12_ = 6.8, *p* = 0.04, η_p_^2^ = 0.53). The FDD of the H/M ratio differed marginally at T60 compared to T0 (frequency × buspirone interaction: *F*_2_,_12_ = 4.6, *p* = 0.07, η_p_^2^ = 0.43). *Post hoc* analysis revealed that the H/M ratio differences between T0 and T60 reached significance at 0.2 Hz (*p* = 0.002), but not at 5 and 10 Hz (*p* = 0.83 and *p* = 0.76, respectively).

## Discussion

The use of the adult decerebrated mouse preparation allowed us to study the effect of buspirone on the H-reflex after acute spinal lesion in a system that was not altered by the presence of anesthetic drugs. The main result of this study was that buspirone had a depressive impact on H-reflex amplitude for the first 20 min after drug administration. This depressive outcome was then attenuated and even reversed to an increased effect on the H-reflex which became significant 40 min post-dose. The absence of reflex variations when the buspirone treatment is given after 5-HT_1__A_ receptor blocking by NAD-299 suggest a participation of this receptor in this observed buspirone activity. Since there is still an even stronger FDD 60 min after buspirone, the observed reflex enhancement later after buspirone is not likely to involve a loss of inhibitory control.

### Buspirone Act as a 5-HT_1__A_ Receptor Agonist on the Reflex

Buspirone is not a pure 5-HT_1__A_ agonist, it also shows some affinity for dopaminergic and other serotoninergic receptors (Loane and Politis, 2012). By using the selective antagonist NAD-299 we show here that 5-HT_1__A_ receptors activation is essential for buspirone induced modulation of H-reflex excitability. Even if this reflex is mainly of monosynaptic nature, it is well known that it remains under the control of several elements (Misiaszek, 2003). The 5-HT_1__A_ receptors can be found at numerous locations on these elements including presynaptic, intrasynaptic and even outside the synaptic innervation. This heterogeneity probably explains why 5-HT have such multiple and opposite effects on motor circuits of the spinal cord, as elegantly reviewed in Perrier and Cotel (2015).

The short-term impact of buspirone observed in our experiments, i.e., reflex reduction, concurs with the literature on the effect of 5-HT_1__A_ agonists. Indeed, monosynaptic reflex reduction has been shown with the 5-HT_1__A_ agonist 8-OH-DPAT in rats with complete spinal lesion under α-chloralose or urethane anesthesia (Nagano et al., 1988; Hasegawa and Ono, 1996a; Honda and Ono, 1999). More recently, buspirone was used as a 5-HT_1__A_ agonist and it was shown that systemic administration in awake humans reduces about 30% of F-wave amplitude, indicating direct decrease in motoneuron excitability and output (D’Amico et al., 2017).

Because 5-HT_1__A_ receptors are mainly present on dorsal laminae of the spinal cord, it was proposed to be also involved in afferent regulation (Giroux et al., 1999; Noga et al., 2009). Such participation in afferent modulation by 5-HT_1__A_ receptors has been confirmed by Crick et al. (1994) in rats (Hasegawa and Ono, 1996b). They showed that monosynaptic reflexes evoked by dorsal root stimulation are depressed by 8-OH-DPAT administration with no change in motoneuronal excitability. This suggests that reflex depression is induced by lowering neurotransmitter release at the presynaptic level. Such afferent regulation could be generated by 5-HT_1__A_ receptors on afferent neurons and could be responsible for increased GABA-mediated inhibition (Gharagozloo et al., 1990).

### The Absence of Anesthesia and Reversal From Inhibitory to Excitatory Effects of Buspirone

A reversal of the effect buspirone (or any other 5-HT_1__A_ agonist) from inhibitory to excitatory later post-treatment has not been reported so far. Such differences with previous experiments could be related to the use of decerebrated preparations and the absence of anesthesia (Meehan et al., 2017) that affect reflex modulation (Ho and Waite, 2002) see also (Schmidt and Jordan, 2000). Indeed, for example, experiments on decerebrated cats disclosed reversal of group I autogenetic inhibition to polysynaptic excitation in extensor motoneurons after exposure to clonidine or L-DOPA, drugs that promote locomotion in spinal cats (Conway et al., 1987; Gossard et al., 1994). Interneurons involved in this reflex reversal are shared with CPGs and supraspinal inputs (Leblond et al., 2000, 2001).

Low threshold stimulation like the one used in the present study might also activate oligosynaptic pathways by some other large-diameter afferent fibers, such as type Ib afferents, that are also in contact with motoneurons. Because such depolarization involves the polysynaptic circuitry, the motor response would have longer delay and may be dissociated from monosynaptic activation (e.g., Figure 1A, bottom trace). Still, long-lasting effects on motoneurons by these pathways are not to be excluded and could be implicated in signal amplitude recorded by EMG.

Indeed, the absence of anesthesia most likely allowed otherwise quiescent spinal networks to be active and participate in the modulation of membrane conductance, affecting motoneuronal responsiveness (Harvey et al., 2006a, b; Li et al., 2006; Murray et al., 2010; see also D’Amico et al., 2014). However, slowly activated currents, like persistent inward currents, required long-duration input and could not be fully actuated by brief stimulations like the ones used in our study (Murray et al., 2011).

It is still not fully clear why the H-reflex was enhanced during the second phase of our experiment and this will be discussed in a later section. Nonetheless, to evaluate if this reversal from inhibition to excitation can be explained by a disinhibition, we measured FDD of the H-reflex. The FDD, also named homosynaptic depression or post-activation depression, was used in many animal models to study spinal reflex disinhibition (Thompson et al., 1992; Yates et al., 2008; Cote et al., 2011; Jeffrey-Gauthier et al., 2019). It was shown that this depression can occurs at higher rate of stimulation without any variation of motoneuronal excitability, reflecting a decreased probability of neurotransmitter release by the activated fibers as a consequence of their repeated activation (Hultborn et al., 1996). Our hypothesis was that disinhibition, in other word a lack of FDD 60 min after buspirone, would explain why there is a higher H-reflex at that moment. Our results showed that FDD is still present 60 min post-buspirone, suggesting that disinhibition would not explain the observed increase of reflex amplitude.

### Opposite Effect of 5-HT_1__A_ According to Receptor Location on the Motoneuron

As mentioned above, 5-HT_1__A_ receptors are located at various locations that can get activated simultaneously. On the one hand, at the synaptic level, 5-HT is responsible for modulating fast-activated potassium channels via 5-HT_1__A_ receptors (Jackson and White, 1990; Penington and Kelly, 1990). It was shown that 5-HT_1__A_ receptors inhibit TASK-1 potassium channels that would contribute to the excitatory effect of 5-HT on spinal motoneurons (Perrier et al., 2003). By lowering outward cation flux, 5-HT_1__A_ receptors shortened the refractory period and facilitated motoneuronal depolarization (Grunnet et al., 2004; Santini and Porter, 2010). This mechanism augments motoneuronal excitability and enhances motor responses to synaptic stimulation. On the other hand, there are extrasynaptic 5-HT_1__A_ receptors that can be activated by spill-over during high 5-HT release at the synaptic level or background concentration of 5-HT (e.g., in systemic administration) that are known to be inhibitory. Indeed, 5-HT_1__A_ receptor stimulation on axon hillocks elicits inhibition of sodium channels that are responsible for initiation of action potentials in motoneurons (Cotel et al., 2013; Perrier et al., 2013; Perrier and Cotel, 2015; Petersen et al., 2016). This inhibition decreases the number of spikes triggered and consequently reduce the amplitude of the EMG.

Thus, when large dose of buspirone is given, as in our experiments, 5-HT_1__A_ receptors inhibit motoneuron output and decrease reflex amplitude through activity at the axon hillock sites even if there is an excitation at the synaptic level. This dual effect of 5-HT_1__A_ receptors on motoneuronal excitability may be involved in the observed biphasic effect of buspirone over time on reflex amplitude through a switch in dominance of receptor type activity.

Indeed, drug action is concentration-dependent, and buspirone pharmacokinetics undergoes a biphasic elimination cycle (Sethy and Francis, 1988). The first half-life of the drug is reached after 24.8 min, a period that matches the transition phase of reflex amplitude in treated animals. This region relies mainly on the participation of astrocytes that have been demonstrated to be involved in 5-HT re-uptake, especially at the extrasynaptic level (Henn and Hamberger, 1971; Ritchie et al., 1981; Kimelberg and Katz, 1985; De-Miguel et al., 2015). Such region-dependent differences in the 5-HT clearance mechanisms could explain the biphasic effect of buspirone on reflex amplitude over time. Moreover, a desensitization of 5-HT_1__A_ receptors after their pharmacological activation have been reported and should be considered as well in that reversal (Seth et al., 1997).

### Reflex Inhibition Concomitant With Excitatory Effect on Locomotion

It was shown in another study from our laboratory that buspirone exerts a considerable acute facilitation of spinally mediated locomotion in mice after a complete mid-thoracic section of the spinal cord (Jeffrey-Gauthier et al., 2018). Indeed, by using the same amount of buspirone than in the present experiments, we were able to trigger locomotion right after the injection in previously paralyzed mice as early as 2 days after a complete lesion. Buspirone was also shown to potentiate locomotion when combined with other treatments (Ung et al., 2012; Gerasimenko et al., 2015). Since we find herein that buspirone have an early depressive effect on the H-reflex, it suggest that locomotion can be triggered during depression of sensorimotor excitability induced by this treatment. This paradox is also observed with the 5-HT_1__A_ partial agonist 8-OH-DPAT, which is known to inhibit the monosynaptic reflex (Nagano et al., 1988; Hasegawa and Ono, 1996a; Honda and Ono, 1999) and can facilitate recovery of locomotor function in spinal rats (Antri et al., 2003, 2005). Sensory inputs provided by the treadmill seem sufficient to initiate and maintain locomotor rhythm with buspirone. The same observation was made in cats where clonidine, a noradrenergic agonist that can trigger locomotion on a treadmill after a complete spinal lesion, reduce reflexes evoked by stimulation of the dorsum of the foot (Barbeau and Rossignol, 1987; Chau et al., 1998a).

These observations with adult animals that walk on a treadmill seem to disagree with results obtained during fictive locomotion in neonatal rodents where 5-HT_1__A_ was reported to have an inhibitory effect on the spinal rhythmic activity (Beato and Nistri, 1998; Liu and Jordan, 2005; Pearlstein et al., 2005; Dunbar et al., 2010). For example, in the brainstem-spinal cord of neonatal mice, 5-HT release during fictive locomotion was enhanced by citalopram, a selective 5-HT re-uptake inhibitor, and a decreased burst duration and amplitude was observed (Dunbar et al., 2010). Since selective 5-HT_1__A_ and 5-HT_1__B_ antagonists reversed the inhibitory effect of citalopram, it was concluded that these receptors may rather be involved in rhythm inhibition. A similar conclusion has been drawn with neonatal rats where blocking 5-HT_1__A/__1__B_ receptors during motor activity, produced by brainstem stimulation, induced speed-up of the rhythm (Liu and Jordan, 2005). In both these studies, locomotor speed was impaired by 5-HT_1__A_ receptor but the alternate pattern of locomotor rhythm was not blocked.

This discrepancy between results obtained during fictive locomotion or locomotion over a treadmill, when there is some exteroceptive stimulation, suggest that sensorimotor control is fundamental to the pro-locomotor effect of buspirone. Many studies employing different methodologies to induce locomotion have disclosed that reflex modulation is associated with locomotor expression (Grillner and Shik, 1973; McCrea, 2001; Frigon et al., 2012). Similarly, buspirone treatment induces spinal reflex re-organization and promotes locomotor activity.

## Conclusion

In summary, even if the role of 5-HT on motoneuron excitability has been extensively studied for more than 50 years, our knowledge is still scarce on how this neuromodulator contribute to sensorimotor control. The heterogeneity of 5-HT receptors locations (pre-, intra- or extra-synaptically) make it really difficult to assess the outcome of a treatment with this neuromodulator after a complete spinal cord injury. Reflecting this heterogeneity, buspirone, if given at a dose that can trigger locomotion, was shown to have biphasic consequence on the H-reflex in time after an acute lesion of the spinal cord, starting with an early and acute inhibition, followed by an excitation of the reflex. This effect seems to be mediated by the activation of 5-HT_1__A_ receptors.

## Data Availability Statement

The datasets generated for this study are available on request to the corresponding author.

## Ethics Statement

The animal study was reviewed and approved by the Comité de bons soins aux animaux.

## Author Contributions

YD designed the study, conducted the experiments, acquired and analyzed the data, and wrote the manuscript. HL conceived and designed the study, acquired and analyzed the data, wrote the manuscript, and obtained funding for this work. Both authors have approved the final version of the manuscript and agreed to be accountable for all aspects of the work.

## Conflict of Interest

The authors declare that the research was conducted in the absence of any commercial or financial relationships that could be construed as a potential conflict of interest.
